# Supplementary material for: Deep learning models for COVID-19 chest x-ray classification: Preventing shortcut learning using feature disentanglement
Source: medRxiv. 2021 Feb 13:2021.02.11.20196766. Preprint. [Version 1] doi: 10.1101/2021.02.11.20196766 (PMC7885941; doi:10.1101/2021.02.11.20196766)
Supplement: Supplement 1 [file media-1.pdf]

Supplementary Information: Deep learning models for COVID-19 chest x-ray classification: Preventing shortcut learning using feature disentanglement

Caleb Robinson[1,+], Anusua Trivedi[1,+], Marian Blazes[3], Anthony Ortiz[1], Jocelyn Desbiens[2], Sunil Gupta[2], Rahul Dodhia[1], Pavan K. Bhatraju[5], W. Conrad Liles[5], Aaron Lee[3,*], Jayashree Kalpathy-Cramer[4], and Juan M. Lavista Ferres[1,*]

[1]Microsoft AI for Good
[2]Intelligent Retinal Imaging Systems
[3]University of Washington
[4]Massachusetts General Hospital
[5]Department of Medicine and Sepsis Center of Research Excellence, University of Washington (SCORE-UW)
[*]Corresponding authors: leeay@uw.edu, jlavista@microsoft.com
[+]these authors contributed equally as first-authors to all academic and professional effects, and their names can be legitimately swapped in their respective publication lists

1 Supplementary related work

Using CXRs for COVID-19. Since the COVID-19 outbreak, various research has tried COVID-19 diagnosis with Convolutional Neural Networks (CNNs) on radiographic images. Many approaches to classify chest x-ray scans to discriminate COVID-19 positive cases have been shown. Focusing on a transfer learning-based approach, (1; 2) compare various classification performances obtained between several popular CNN architectures. A similar approach employed by (3) uses Resnet-based architectures with a 5-fold cross-validation strategy. (4) propose a novel CNN architecture for the COVID classification task. However, all this research relies on the open-source COVID-CHESTXRAY dataset (5), made up of COVID-19+ CXRs sourced from around the web. For COVID-19 negative cases, data are typically sampled from other open CXR datasets. However, if any bias is present within these datasets, the model could learn the underlying biases, rather than learning COVID-19 related features. For instance, a model could potentially learn to discriminate based on differences due to the scanning devices, or unique windowing parameters of each CXR, or some other acquisition settings. This can result in the classification task yielding apparently optimal classification performance. Domain adaptation techniques like feature disentanglement can be useful to address this issue.

Domain Adaptation. Domain adaptation (DA) transfers the knowledge learned from one or more source domains to a target domain. Discrepancy-based DA approaches (6; 7; 8; 9), adversary-based approaches (10; 11; 12), and reconstruction-based approaches (13; 14; 15; 16) are designed

to handle a single-source to single-target adaptation. Originating from the theoretical analysis in (17; 18; 19), the multiple source domain adaptation (MSDA) approach assumes that training data are collected from multiple sources and has been applied to several practical applications (20; 21; 22) (17) introduces an approach with HΔH-divergence between the weighted combination of source domains and a target domain.

Disentangled Representation Learning. Disentangled representations learning tries to model the factors of knowledge variation. (23; 24; 12; 25) aims at learning an interpretable representation using generative adversarial networks (GANs) (26; 27) and variational autoencoders (VAEs) (28; 29). (30) proposes to disentangle the feature representation into a domain-invariant content space and a domain-specific attribute space in a fully supervised setting. (25) proposes an auxiliary classifier GAN (AC-GAN) to achieve representation disentanglement. However, all these approaches specialize in disentangling representation in a single domain. (12) introduces a unified feature disentangler for domain-invariant representation from data across multiple domains. However, they assume multiple source domain availability during training, which limits its application.

References

[1] P. K. Sethy and S. K. Behera, "Detection of coronavirus disease (COVID-19) based on deep features," *Preprints*, 2020.

[2] I. D. Apostolopoulos and T. A. Mpesiana, "COVID-19: automatic detection from X-ray images utilizing transfer learning with convolutional neural networks," *Physical and Engineering Sciences in Medicine*, p. 1, 2020.

[3] A. Narin, C. Kaya, and Z. Pamuk, "Automatic detection of coronavirus disease (COVID-19) using X-ray images and deep convolutional neural networks," *arXiv preprint arXiv:2003.10849*, 2020.

[4] Z. Q. L. Linda Wang and A. Wong, "COVID-Net: A tailored deep convolutional neural network design for detection of COVID-19 cases from chest radiography images," *arXiv preprint arXiv:2003.09871*, 2020.

[5] J. P. Cohen, P. Morrison, and L. Dao, "COVID-19 image data collection," *arXiv preprint 2003.11597*, 2020.

[6] M. Long, H. Zhu, J. Wang, and M. I. Jordan, "Deep transfer learning with joint adaptation networks," in *International conference on machine learning*, pp. 2208–2217, PMLR, 2017.

[7] E. Tzeng, J. Hoffman, N. Zhang, K. Saenko, and T. Darrell, "Deep domain confusion: Maximizing for domain invariance," *arXiv preprint arXiv:1412.3474*, 2014.

[8] M. Ghifary, W. B. Kleijn, and M. Zhang, "Domain adaptive neural networks for object recognition," in *Pacific Rim international conference on artificial intelligence*, pp. 898–904, Springer, 2014.

[9] X. Peng and K. Saenko, "Synthetic to real adaptation with generative correlation alignment networks," in *2018 IEEE Winter Conference on Applications of Computer Vision (WACV)*, pp. 1982–1991, IEEE, 2018.

[10] M.-Y. Liu and O. Tuzel, “Coupled generative adversarial networks,” in *Advances in neural information processing systems*, pp. 469–477, 2016.

[11] E. Tzeng, J. Hoffman, K. Saenko, and T. Darrell, “Adversarial discriminative domain adaptation,” in *Proceedings of the IEEE conference on computer vision and pattern recognition*, pp. 7167–7176, 2017.

[12] A. H. Liu, Y.-C. Liu, Y.-Y. Yeh, and Y.-C. F. Wang, “A unified feature disentangler for multi-domain image translation and manipulation,” in *Advances in neural information processing systems*, pp. 2590–2599, 2018.

[13] Z. Yi, H. Zhang, P. Tan, and M. Gong, “Dualgan: Unsupervised dual learning for image-to-image translation,” in *Proceedings of the IEEE international conference on computer vision*, pp. 2849–2857, 2017.

[14] J.-Y. Zhu, T. Park, P. Isola, and A. A. Efros, “Unpaired image-to-image translation using cycle-consistent adversarial networks,” in *Proceedings of the IEEE international conference on computer vision*, pp. 2223–2232, 2017.

[15] J. Hoffman, E. Tzeng, T. Park, J.-Y. Zhu, P. Isola, K. Saenko, A. Efros, and T. Darrell, “Cycada: Cycle-consistent adversarial domain adaptation,” in *International conference on machine learning*, pp. 1989–1998, PMLR, 2018.

[16] T. Kim, M. Cha, H. Kim, J. K. Lee, and J. Kim, “Learning to discover cross-domain relations with generative adversarial networks,” *arXiv preprint arXiv:1703.05192*, 2017.

[17] S. Ben-David, J. Blitzer, K. Crammer, A. Kulesza, F. Pereira, and J. W. Vaughan, “A theory of learning from different domains,” *Machine learning*, vol. 79, no. 1-2, pp. 151–175, 2010.

[18] Y. Mansour, M. Mohri, and A. Rostamizadeh, “Domain adaptation with multiple sources,” in *Advances in neural information processing systems*, pp. 1041–1048, 2009.

[19] K. Crammer, M. Kearns, and J. Wortman, “Learning from multiple sources,” *Journal of Machine Learning Research*, vol. 9, no. Aug, pp. 1757–1774, 2008.

[20] R. Xu, Z. Chen, W. Zuo, J. Yan, and L. Lin, “Deep cocktail network: Multi-source unsupervised domain adaptation with category shift,” in *Proceedings of the IEEE Conference on Computer Vision and Pattern Recognition*, pp. 3964–3973, 2018.

[21] L. Duan, D. Xu, and S.-F. Chang, “Exploiting web images for event recognition in consumer videos: A multiple source domain adaptation approach,” in *2012 IEEE Conference on Computer Vision and Pattern Recognition*, pp. 1338–1345, IEEE, 2012.

[22] X. Peng, Q. Bai, X. Xia, Z. Huang, K. Saenko, and B. Wang, “Moment matching for multi-source domain adaptation,” in *Proceedings of the IEEE International Conference on Computer Vision*, pp. 1406–1415, 2019.

[23] M. F. Mathieu, J. J. Zhao, J. Zhao, A. Ramesh, P. Sprechmann, and Y. LeCun, “Disentangling factors of variation in deep representation using adversarial training,” in *Advances in neural information processing systems*, pp. 5040–5048, 2016.

[24] A. Makhzani, J. Shlens, N. Jaitly, I. Goodfellow, and B. Frey, "Adversarial autoencoders," *arXiv preprint arXiv:1511.05644*, 2015.

[25] A. Odena, C. Olah, and J. Shlens, "Conditional image synthesis with auxiliary classifier gans," in *International conference on machine learning*, pp. 2642–2651, 2017.

[26] I. Goodfellow, J. Pouget-Abadie, M. Mirza, B. Xu, D. Warde-Farley, S. Ozair, A. Courville, and Y. Bengio, "Generative adversarial nets," in *Advances in neural information processing systems*, pp. 2672–2680, 2014.

[27] D. P. Kingma, S. Mohamed, D. J. Rezende, and M. Welling, "Semi-supervised learning with deep generative models," in *Advances in neural information processing systems*, pp. 3581–3589, 2014.

[28] D. Jimenez Rezende, S. Mohamed, and D. Wierstra, "Stochastic backpropagation and approximate inference in deep generative models," *arXiv*, pp. arXiv–1401, 2014.

[29] D. P. Kingma and M. Welling, "Auto-encoding variational bayes," *arXiv preprint arXiv:1312.6114*, 2013.

[30] H.-Y. Lee, H.-Y. Tseng, J.-B. Huang, M. Singh, and M.-H. Yang, "Diverse image-to-image translation via disentangled representations," in *Proceedings of the European conference on computer vision (ECCV)*, pp. 35–51, 2018.
